# Analysis of associations between dietary patterns, genetic disposition, and cognitive function in data from UK Biobank

**DOI:** 10.1007/s00394-022-02976-y

**Published:** 2022-09-24

**Authors:** Christina-Alexandra Schulz, Leonie Weinhold, Matthias Schmid, Markus M. Nöthen, Ute Nöthlings

**Affiliations:** 1 Institute of Nutrition and Food Sciences, Nutritional Epidemiology, University of Bonn, Bonn, Germany; 2 Department of Medical Biometry, Informatics and Epidemiology, University Hospital Bonn, University of Bonn, Bonn, Germany; 3 Institute of Human Genetics, University of Bonn, School of Medicine and University Hospital Bonn, Bonn, Germany

**Keywords:** Cognition, Fluid intelligence, General cognitive function, Dietary pattern, Genetic disposition, Polygenic score

## Abstract

**Purpose:**

Research suggests that diet influences cognitive function and the risk for neurodegenerative disease. The present study aimed to determine whether a recently developed diet score, based on recommendations for dietary priorities for cardio metabolic health, was associated with fluid intelligence, and whether these associations were modified by individual genetic disposition.

**Methods:**

This research has been conducted using the UK Biobank Resource. Analyses were performed using self-report data on diet and the results for the verbal-numerical reasoning test of fluid intelligence of 104,895 individuals (46% male: mean age at recruitment 57.1 years (range 40–70)). For each participant, a diet score and a polygenic score (PGS) were constructed, which evaluated predefined cut-offs for the intake of fruit, vegetables, fish, processed meat, unprocessed meat, whole grain, and refined grain, and ranged from 0 (unfavorable) to 7 (favorable). To investigate whether the diet score was associated with fluid intelligence, and whether the association was modified by PGS, linear regression analyses were performed.

**Results:**

The average diet score was 3.9 (SD 1.4). After adjustment for selected confounders, a positive association was found between baseline fluid intelligence and PGS (*P* < 0.001). No association was found between baseline fluid intelligence and diet score (*P* = 0.601), even after stratification for PGS, or in participants with longitudinal data available (*n* = 9,482).

**Conclusion:**

In this middle-aged cohort, no evidence was found for an association between the investigated diet score and either baseline or longitudinal fluid intelligence. However, as in previous reports, fluid intelligence was strongly associated with a PGS for general cognitive function.

**Supplementary Information:**

The online version contains supplementary material available at 10.1007/s00394-022-02976-y.

## Introduction

Elucidating the role of dietary intake in cognitive function, cognitive decline, and neurodegenerative disease development is important in terms of disease prevention. Research has shown that nutrition influences many of the molecular mechanisms that underlie cognitive function, such as neurogenesis, synaptic plasticity, and neuronal connectivity [1–3]. However, studies of the association between cognitive function, cognitive decline, and/or the risk of dementia and dietary patterns, such as the Mediterranean, Nordic, and DASH (Dietary Approaches to Stop Hypertension) diets, have generated inconclusive results [4–11]. Only the *Mediterranean-Dietary Approaches to Stop Hypertension Diet Intervention for Neurodegenerative Delay* (MIND) diet has consistently shown beneficial associations in terms of the risk of dementia and cognitive decline [12, 13]. However, a more consistent research finding is that dietary habits influence cardiometabolic risk factors, including blood pressure, glucose-insulin homeostasis, lipoprotein concentrations and function, and inflammation [14]. Interestingly, several studies have demonstrated that a higher risk of cognitive impairment in later life is associated with cardiovascular risk factors during middle age [15–18]. Based on recommendations for dietary priorities for cardio metabolic health [14], Lourida et al. constructed a diet score as one component of a lifestyle score [19]. Analyses based on the population-based UK Biobank showed that participants with an unfavorable lifestyle had a higher risk of dementia over an 8 year period of follow-up.

Cognitive function incorporates multiple domains and abilities. One cognitive function domain is fluid intelligence, which refers to the capacity for reasoning and novel problem-solving [20]. Research has shown that declines in the ability to live and function independently as a person correlates highly with decline in fluid intelligence [21, 22]. Hence, an improved understanding of the determinants of fluid intelligence is warranted [19, 23].

As with dementia, cognitive functions also show a substantial degree of heritability [24]. Heritability estimates for cognitive function range between 12 and 25% [25–27], and recent genome-wide association studies (GWAS) of general cognitive function have identified more than 140 associated loci [24–27].

To our knowledge, no study to date has investigated whether: (1) the diet score of Lourida et al. [19] is associated with fluid intelligence; or (2) genetic disposition for cognitive function alters the association between diet and fluid intelligence. Therefore, the aim of the present study was to determine whether the “healthy diet” score [19] was associated with baseline fluid intelligence and change in fluid intelligence over time in the UK Biobank cohort. To test whether this association was influenced by individual genetic disposition, a polygenic score (PGS) approach was applied, which takes into consideration the polygenic nature of complex traits [28]. A score comprising of a set of single-nucleotide polymorphisms (SNPs) associated with general cognitive function [26] was used.

## Research design and methods

### The UK Biobank study

This research has been conducted using the UK Biobank Resource. Individual-level data for the present analyses were drawn from the UK Biobank project under application number 31615 “Genetic factors as a biological link between food intake and cognition”. UK Biobank was established to allow detailed longitudinal investigations of the genetic and nongenetic determinants of the diseases of middle and old age [29, 30]. The UK Biobank cohort comprises > 500,000 participants aged 40–69 years at the time of recruitment 55% female). All participants were recruited from the general population. Recruitment invitations were mailed to 9 million individuals, whose contact details were obtained from National Health Service central registers [29].

The ethical approval process for the UK Biobank study is described elsewhere [31].

Baseline assessments were conducted between 2006 and 2010 at a total of 22 assessment centers across the United Kingdom [32]. Here, participants completed a self-report touch-screen questionnaire comprising items on sociodemographic characteristics, general health, medical history, and dietary intake and other lifestyle exposures. During the baseline visit, first, the touch-screen questionnaire was administrated, which was immediately followed by the cognitive function tests [33]. Follow-up examinations commenced in 2012. To date, these have comprised: (1) the first repeat assessment visit (2012–13), (2) the first imaging visit (2014 +), and (3) the first repeat imaging visit (2019 +). The touch-screen questionnaire and other resources can be found on the UK Biobank webpage [32]. Dietary data were assessed in a short touch-screen questionnaire data, which included information on the frequency of the consumption of 29 main foods and food items over the preceding year. At baseline, cognitive functioning was assessed at the assessment using a 15 min self-administered computerized battery, which was developed specifically for the UK Biobank study to enable population-scale cognitive testing that could be administered without researcher supervision [34, 35].

Several of the cognitive function tests administered at the baseline visit were later re-implemented as web-based questionnaires [36]. Hence, from 2014, participants were invited to complete these online tests at home rather than at an assessment center [34]. Fluid intelligence was assessed using the Verbal-Numerical Reasoning (VNR) test [37]. The VNR-test comprises 13 multiple-choice questions that assess verbal and numerical problem-solving. The VNR-test score indicates the number of correct responses achieved within 2 min (range 0–13).

At the baseline UK Biobank assessments, data on lifestyle, including dietary intake, and genotype were obtained for a total of 501,599 and 500,000 participants, respectively [38]. Baseline VNR-test data were available for *n* = 165,456 participants [39]. A total of 13,470 participants completed the VNR-test at baseline and at a minimum of one follow-up visit.

### Present study population

**Fig. 1:**
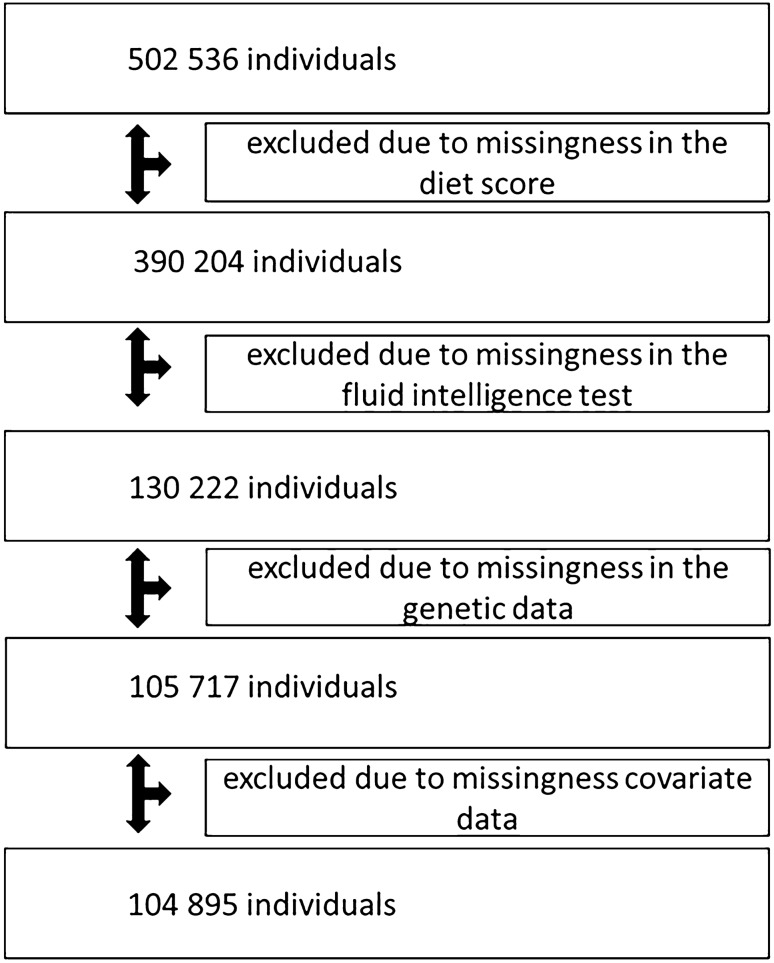
Flowchart of the participants included in the analysis

The present analyses were performed using data from 104,895 UK Biobank participants (Fig. 1). Inclusion criteria were the availability of data: on (1) dietary intake, (2) fluid intelligence, and (3) genetic factors. Exclusion criteria were: (1) self-reported ancestry other than “British”, “White”, “Irish”, or “any other white background”; (2) close kinship (third degree or closer relationship with an already recruited participant); (3) missing genetic data; (4) failed quality control (QC) of the genetic data; and (5) missing data on covariates. The analysis of change in fluid intelligence between baseline and the respective follow-up examination/s was performed in participants for whom data from any of the follow-up visits (2012–13; 2015 + , and/or 2019 +) were available (*n* = 9482 participants).

### Diet score

To assess whether dietary patterns were associated with fluid intelligence, a recently developed diet score of Lourida et al. [19] was used. The diet score incorporates seven components (i.e., fruits, vegetables, fish, processed meats, unprocessed red meats, whole grain, and refined grain). For each participant, the diet score was calculated by summing the points for each of the seven food components (range 0–7 points). In accordance with the predefined cut-offs of Lourida et al. [19], fulfillment was scored with 1 point, and non-fulfillment with 0 points (Table 1). A higher score indicates a more favorable diet. For the purposes of the present analyses, three diet score categories were defined: low (0–1 point), intermediate (2–5 points), and high (6–7 points) category.

**Table 1:** Components and their cut-offs, included in the diet score^a,b^

Diet component	Cut-off^c^	Point
Fruits’ intake	≥ 3 servings/day	1
Vegetables’ intake	≥ 3 servings/day	1
Fish intake	≥ 2 servings/week	1
Processed meat intake	≤ 1 servings/week	1
Unprocessed red meat intake	≤ 1.5 servings/week	1
Whole grain intake	≥ 3 servings/day	1
Refined grains’ intake	≤ 1.5 servings/day	1

^a^The score was previously developed by Lourida et al. [19]

^b^The points of the individual components were summed up. The total diet score reached from 0 to 7

^c^An intake in accordance with the cutoff was scored with one point

### Genetic data

The imputed (reference: Haplotype Reference Consortium [40]) genetic data were downloaded from the server of UK Biobank [38]. QC was performed for the genetic data of all 104,895 participants included in the present study. Here of all available 93,095,623 genetic markers, all variants with an imputation quality of less than 0.6 were removed (*n* = 90,078,127). Moreover, SNPs with missing genotype information > 0.03 (*n* = 187,280), when deviating from Hardy–Weinberg equilibrium (HWE > 0.000001; *n* = 13,386) or if they were rare variants (minor allele frequency (MAF) < 0.01; *n* = 1,919,230) were removed as well. This led to a total number of 897,600 genetic variants available in 104,895 participants that were used to construct the PGS. The PGS was constructed by first, clumping the SNPs to capture those that have the lowest *p* values (based on the GWAS of Davies et al.[26]) in a linkage disequilibrium block (*r*^2^ = 0.2, range 1000 kb).

### Polygenic score for general cognitive function

Individual genetic disposition for cognitive function was assessed using a PGS approach [28]. For each participant, a PGS was calculated based on common variants discovered in a previous GWAS of general cognitive function in individuals of European ancestry [26]. The PGS was calculated by summing the effect size-weighted number of associated alleles for each participant. For analysis, we used the PGS including all SNPs with *p* values < 0.2 based on visual inspection of the *r*-squared value of the crude VNR-score–PGS association. Finally, for each participant, the PGS was *z*-standardized, and then categorized according to genetic disposition for a high score on the fluid intelligence test using the categories low (lowest quintile), intermediate (quintile 2–4), and high (highest quintile).

### Statistical analysis

The baseline characteristics of the study population are presented using means and standard deviations (SD) for continuous variables, and counts and percentages for categorical variables.

Linear regression models were used to test the association between fluid intelligence (i.e., results of the VNR-test) and both the diet score and the PGS. Results obtained from linear regression models are presented in terms of coefficient estimates (beta) with 95% confidence intervals (CI). To adjust the multiple linear regression models, four confounders were considered: age, sex, educational status, and socio economic status (SES). Educational status was categorized as: higher (college or university degree/other professional qualifications e.g., nursing, teaching); upper secondary (A levels/AS levels or equivalent); lower secondary (O levels/ General Certificate of Secondary Educations or equivalent/Certificate of Secondary Education or equivalent); vocational (National Vocational Qualifications or Higher National Diploma or Higher National Certificate or equivalent); or other. SES was assessed using the Townsend deprivation index, which combines information on social class, employment, car ownership, and housing [41]. In accordance with the calculated quintile of the Townsend deprivation index, SES was categorized as low (quintile 1), intermediate (quintiles 2–4), or high (quintile 5). First, univariable regression models were used. Second, all models were adjusted for age, sex, educational status, and the Townsend deprivation index. In addition, models including the PGS were adjusted for the first 20 principal components (PC) of ancestry. To present a measure for effect size, Cohen’s *f*^2^ was calculated. As a rule of thumb, *f*^2^ = 0.02 indicates a small, *f*^2^ = 0.15 a medium, and *f*^2^ = 0.35 a large effect [42]. In addition, the analyses were repeated using the continuous diet score and the continuous PGS, rather than the categorized diet score and categorized PGS. Furthermore, each of the seven dietary components was analyzed separately in univariate and adjusted linear regression models, as described above. Additionally, in participants for whom longitudinal data were available, change in fluid intelligence between baseline and the respective follow-up examination/s was analyzed by linear mixed models, with the participant ID being considered as a random effect to account for intra individual dependencies. The linear mixed models were adjusted for the baseline results of the VNR-test, age, sex, educational status, and SES. Analyses were performed to test whether individual genetic disposition modifieds the association between the diet score and performance in the VNR-test by introducing an interaction term (diet score × PGS) into the model, and analyses were stratified according to PGS category. A post hoc analysis was then performed to investigate whether the results were impacted by adjustment for further covariates, (i.e., a: alcohol intake, smoking, physical activity, and b: body mass index (BMI). *P* values were 2-sided. Statistical significance was set at < 0.05. All analyses were performed using the R Software for Statistical Computing, version 3.6.1 [43].

## Results

### Characteristics of the present cohort at the baseline UK Biobank visit

The mean age of the 104,895 participants at the baseline visit was 57.1 (SD 8.0) years. The majority of the cohort was female (54%) (Table 2). Almost half of the cohort had a higher education status (49.3%). Approximately one-fifth of the participants were classified, respectively, in the lowest or the highest SES quintile (least deprived 21.1%; most deprived 18.2%). In the VNR-test, the average number of correct responses was 6.1 (SD 2.1). The average diet score was 3.9 (SD 1.4). The majority of participants reported that their consumption of each of the individual diet score components was in line with the cut-offs predefined by Lourida et al. [19] (fruit: 53%; vegetables: 87%; fish: 54%; processed meat: 68%; unprocessed meat: 51%; refined grain: 69%). Nonetheless, only 9% of the participants reported the consumption of 3 servings of whole grain per day. Overall, the lifestyle of the participants was favorable, as almost 91% of the participants reported that they were current non-smokers, and 84% reported that they engaged in regular physical activity. Table 2 provides an overview of the baseline characteristics of the cohort. The PGS showed an approximately normal distribution (data not shown).

**Table 2:** Characteristics of the 104,895 participants from the UKBiobank

Characteristic	Mean/*n*	SD/%
Age (years)	57.1	8.0
Sex (male)	48,239	46.0
VNR-test (correctly answered questions)	6.1	2.1
Education		
Higher	51,715	49.3
Upper secondary	8000	7.6
Lower secondary	23,617	22.5
Vocational	6242	6.0
Other	15321	14.6
Socioeconomic status quintile		
1 (least deprived)	22,105	21.1
2–4	63,738	60.8
5 (most deprived)	19,052	18.2
Lifestyle factors		
No current smoking	95,067	90.9
Regular physical activity	84,211	83.9
Moderate alcohol consumption	70,332	67.1
Diet score	3.9	1.4
Favorable dietary intake		
Fruits (≥ 3 servings/day)	55,580	53.0
Vegetables (≥ 3 servings/day)	90,845	86.6
Fish (≥ 2 servings/week)	56,278	53.7
Processed meat (≤ 1 servings/week)	71,296	68.0
Unprocessed red meat (≤ 1.5 servings/week)	53,184	50.7
Whole grain (≥ 3 servings/day)	9910	9.4
Refined grains (≤ 1.5 servings/day)	72,657	69.3

*SD* standard deviation, *PGS* polygenic risk score, *VNR-test* verbal-number-reasoning test

### Association between fluid intelligence and diet score

In the unadjusted model, the diet score was positively associated with performance in the VNR-test (*P* < 0.0001). However, the magnitude of the effect was small (Cohen’s *f*^2^ = 0.001), since for participants with a high diet score, the estimated average difference in the number of correct responses was only 0.25 (95% CI 0.19–0.32) compared to participants with a low diet score. The association did not remain after adjustment for further confounders. After adjustment for age, sex, educational status, and the Townsend deprivation index, participants with an intermediate or a high diet score did not achieve more correct responses at baseline (*P* = 0.601) than participants with a low diet score (Table 3 and S1). In the adjusted model, no association was found between the continuous diet score and the VNR-test (beta = − 0.001; 95% CI − 0.01 − 0.01; Cohen’s *f*^2^ < 0.001, *P* = 0.780). Interestingly, however, slightly different effects were observed for the individual components of the diet score. In the basic model, that a higher intake of either fruits, vegetable, fish, or processed meat was associated with lower cognitive performance, while a higher intake of unprocessed meat, whole grain, or refined grain was associated with a lower cognitive performance. Whereas fruit intake was no longer associated with cognitive performance after adjustment (beta = − 0.01, CI − 0.03 − 0.02, *P* = 0.64), the associations for vegetables, fish, processed meat, unprocessed meat, whole grain, and refined grain remained (beta = − 0.04, CI − 0.07 to − 0.003, *P* = 0.03; beta = − 0.09, CI − 0.11 to − 0.06 *P* < 0.0001; beta = − 0.11, CI − 0.13 to − 0.08, *P* < 0.0001; beta = 0.02, CI 0.0001–0.57, *P* = 0.04; beta = 0.15, CI 0.11–0.19, *P* < 0.0001; and beta = 0.14, CI 0.11–0.16, *P* < 0.0001, respectively). Results of the analysis of the individual components of the diet score are provided in the Supplement (Table S2).

**Table 3:** Association between VNR-test and diet score, or the PGS, respectively, in 104,895 participants from the UK Biobank

	Low	Intermediate	High	*P* value	Cohen’s *f*^2^
Diet score	0 [Reference]	0.16 (0.11–0.22)	0.25 (0.19–0.32)	2.83e-14	< 0.001
Diet score^a^	0 [Reference]	− 0.01 (− 0.06 − 0.04)	− 0.02 (− 0.08 − 0.04)	0.601	< 0.001
PGS^b^	0 [Reference]	1.55 (1.53–1.58)	3.36 (3.32–3.39)	< 10e-70	0.338
PGS^c^	0 [Reference]	1.30 (1.27–1.32)	2.88 (2.84–2.91)	< 10e-70	0.262

Coefficient estimates (95% CI) and *P* values were obtained from linear regression models

*PGS* polygenic risk score, *VNR-test* verbal-number-reasoning test

^a^Adjusted for age, sex, education, and the Townsend deprivation index

^b^Adjusted for PC 1–20

^c^Adjusted for age, sex, education, the Townsend deprivation index, and PC 1–20

### Association between fluid intelligence and the PGS

A positive association was found between the VNR-test at baseline and the PGS (Cohen’s *f*^2^ = 0.262). A similar increase in the number of correct responses was observed across all PGS categories (*P* < 0.0001). After adjustment for age, sex, educational status, the Townsend deprivation index, and the first 20 PCs, the estimated difference in the number of correct responses was 1.30 (95% CI 1.27–1.32) for participants with an intermediate PGS compared to participants with a low PGS. Similarly, on average, participants with a high PGS had three more correct responses on the baseline VNR-test (2.88; 95% CI 2.84–2.91) than participants with a low PGS (Table 3). Notably, these findings replicate those of a previous analysis conducted using data from the UK Biobank [26]. There was no evidence that the individual genetic disposition modifies the association between the diet score and performance in the VNR-test in the fully adjusted model (*P*_-interaction_ = 0.051). No association was found between the diet score and performance in the VNR-test in any of their PGS strata (Table S4).

### Fluid intelligence in relation to the diet score and genetic disposition

**Fig. 2:**
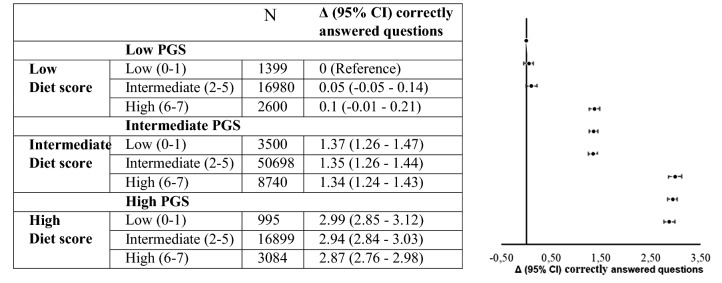
Association between the VNR-test diet and the PGS in 104,895 participants from the UK Biobank. Coefficient estimates and *P* values were obtained from linear regression models adjusted for age, sex, education, the Townsend deprivation index, and PC 1–20. *PC* principal component, *PGS* polygenic risk score, *VNR-test* verbal-number-reasoning test

A model including both the diet score and the PGS was analyzed. The findings were comparable to those obtained in the analysis of fluid intelligence and either the diet score or the PGS alone. Taking the low PGS and low diet score categories as the reference participants, more correct responses in the VNR-test were observed among participants classified in the intermediate (around 1.3 more correct responses) and high (around 2.9 more correct responses) PGS strata, but no difference in VNR-test score was observed between the strata of the diet score. The number of correct responses in the VNR-test was comparable across the three diet score groups (low: 0–0.1, intermediate 1.34–1.37, and high 2.87–2.99) (Fig. 2).

### Change in fluid intelligence over time

For 9482 of the 104,895 participants, longitudinal data on fluid intelligence were available. These participants had attended follow-up visits at an average of 3.0 years (SD 0.3, first follow-up; *n* = 3,297) and 7.3 years (SD 1.3, second follow-up; *n* = 7,349) after the baseline UK Biobank visit. For the VNR-test, the mean change in the number of correct responses was 0.19 (baseline to first follow-up) and 0.03 (baseline to second follow-up). Thus, participants achieved slightly more correct responses when the test was completed a second or third time. However, change in VNR-test performance was similar in participants with a high diet score compared to those with a low diet score (*P* = 0.4227) (Table S3). Moreover, the inter-PGS group differences in VNR-test results remained constant over time (*P*_Interaction_ = 0.2016).

### Post hoc analysis

After adjustment for additional confounders (i.e., a alcohol intake, smoking, physical activity, and b: BMI) the results remained virtually unchanged (Table S4).

## Discussion

The present analysis found no evidence that a diet score was associated with fluid intelligence. Furthermore, no evidence was found for the influence on the relationship between diet score and fluid intelligence of a PGS for general cognitive function [26]. Similar findings were observed in the investigation of the association with change in fluid intelligence over on average 6 year (range 2.1–9.7) period of follow-up.

The present results contrast with those of several previous observational studies involving both short and long follow-up periods [7, 9, 13, 44, 45], which reported modest associations between dietary patterns and cognitive health. A study from France found that adherence to a Mediterranean-type dietary pattern was associated with a less pronounced decline in performance in the Mini- Mental State examination (MMSE) [7]. Similarly, a study from Sweden found that a Western diet was associated with greater cognitive decline, while a prudent diet was associated with less cognitive decline [44]. In the same cohort, the Nordic diet was associated with a superior preservation of cognitive function [9]. Furthermore, a study in the US showed that the MIND-Diet was significantly associated with a slower decline in cognitive abilities [13]. Similarly, results from the Singapore Chinese Health Study showed that adherence to a “healthy dietary pattern” in midlife was associated with a lower risk for cognitive impairment in later life, as measured using a “Singapore-modified” MMSE [45]. In contrast, the present findings are consistent with the results of two previous long-term follow-up studies. In the Atherosclerosis Risk in Communities study, a 20-year change in global cognitive function was assessed using three cognitive tests at three time points across the follow-up period. The authors found no association between a Western and a prudent dietary pattern in midlife [46]. Similar findings were obtained in the Rancho Bernardo Study: whereas the MED diet was associated with superior global cognitive function at baseline, no association with cognitive decline over time was found for the alternate MED, the AHEI-2010, or a dietary pattern derived from factor analysis [47]. With the exception of one report [45], these previous studies investigated older individuals, whose average baseline ages range between ≥ 60 [44] and 81 years [13]. In contrast, the present study investigated younger participants (mean baseline age 57.1). Given that cognitive functioning declines with age, this may explain cross-study inconsistencies. Indeed, even in the UK Biobank, research has shown a significant correlation between performance in all of the administered cognitive tests and age. Older individuals in the UK Biobank showed a poorer performance in all but one test (absolute Pearson correlations for the respective test and age ranged from 0.16 to 0.60, *P* ≤ 0.040) [48]. Moreover, a previous study found a weak but significant negative association between age and fluid intelligence across three time points in UK Biobank participants [21]. Cornelis et al. showed that together with other cognitive functions, baseline fluid intelligence was lower in participants aged 65 + years compared to participants aged < 45 years. However, the authors concluded that declines in cognitive abilities below the age of 65 years are small [34]. Further potential explanations for the observed cross-study inconsistencies may include differences in terms of age and sex distributions, duration of follow-up, the investigated dietary patterns, and the dietary instruments used to obtain the dietary data. A further potential explanation for the observed cross-study inconsistencies is selection bias [49]. Previous authors have proposed the existence in the UK Biobank cohort of a “healthy volunteer” selection bias, since the participants were less likely to be obese, to smoke, and to drink alcohol on a daily basis, and had fewer self-reported health conditions compared with individuals from the general population. Research has found that on average, UK Biobank participants are more health-conscious than individuals from the general population [49]. This may explain the fact that a fairly high proportion of participants were current non-smokers (91%), engaged in regular physical activity (84%), or reported only a moderate level of alcohol consumption (67%) (Table 2). Furthermore, the present analysis included only around 20% (*n* = 104,895) of the 502,536 UK Biobank participants, and only 9% (*n* = 9482) of these 104,895 participants could be included in the longitudinal analyses due to limited data availability. The largest missing data rate was found for the present outcome variable (i.e., the VNR-test score), since this test was only used at 10 of the recruitment sites [34]. Previous authors have also suggested that measurement errors—which would contribute to type 2 errors—may be of concern for the cognitive tests conducted in the UK Biobank, and have pointed out that the issue of whether measurement error was random or varied according to age or time of recruitment remains unclear[34].

Two specific aspects of the present analyses warrant further discussion: the strongly reduced (and no longer significant) association for the diet score after adjustment for confounding factors; and the associations between the VNR-score and the individual dietary components. With respect to the latter, a recently published study using the UK Biobank resource observed similar associations between individual dietary components and a more comprehensive cognitive outcome than that used in the present analysis [11]. Here, Hepsomali and Groeger reported an inverse association between vegetable intake and a principal component analysis (PCA) derived score for general cognitive ability [11]. In general, previous research has established that both educational status [50] and SES [51] are associated with cognitive function. Therefore, an alteration in the effect of the diet score was expected and confirmed in the fully adjusted model in our study (Table S1, Table 3). Interestingly, slightly different attenuations after adjustments were observed for the individual components of the diet score. This could indicate the existence of differences in the association with fluid intelligence between the separate diet components, and in their interplay with the investigated covariates. Yet, in the present cohort, descriptive analyses of the covariates revealed a balanced distribution for educational status, SES, and sex for the individual dietary components, and in the regression models, the estimated effects of the covariates were almost identical (data not shown). Thus, these covariates may not explain the differences in the associations of the individual food groups that were observed in the multivariate models.

The present finding that individual genetic disposition did not influence the association between the diet score and the VNR-test is consistent with the recent study conducted by Lourida et al. [19] which found that a favorable lifestyle was associated with a lower risk of dementia independent of the genetic risk [19]. However, the authors did not report whether the diet score—which was included as one component of the lifestyle score—was itself associated with risk for incident dementia. In contrast, the population-based Rotterdam cohort study found that a healthy diet score, as based on adherence to the 2015 Dutch dietary guidelines, associated with a lower risk for dementia in participants with a low and intermediate genetic risk but not in participants with a high genetic risk [23]. Previous studies have generated inconsistent results concerning the role of genetic disposition for Alzheimer disease, i.e., the *APOE ε4* genotype. In one study, the association between velocity of cognitive decline and different nutrient patterns varied depending on the *APOE ε4* genotype [52]. Another study found that *APOE ε4* carriers, but not *APOE ε4* non-carriers, showed slower rates of decline in global cognition and in multiple cognitive domains in relation to n-3 fatty acid and seafood consumption [53]. In contrast, no effect of the *APOE ε4* genotypes was found in two other studies [54, 55]. Notably, the *APOE ε4* genotype was included in the PGS used, because it has been associated with general cognitive function in the most recent GWAS [26].

One potential explanation for the observed cross-study differences is the heterogeneity of the applied tests of cognitive function. Results from a validation study of 160 participants showed that among other measures of cognition used in UK Biobank, performance in the fluid intelligence test correlated well (Pearson correlation *r* > 0.55) with a general measure of cognitive ability that was based on a battery of standard neuropsychological tests. The authors suggest that the UK Biobank tests, including the fluid intelligence test, load strongly on general cognitive ability [48]. Hence, the VNR-test administrated in the UK Biobank seems to be a reliable and valid measure of cognitive performance. Although all cognitive tests correlate positively, whereby an individual who performs well on one test will tend to perform well on others [24, 56], they do not measure the same ability. As discussed elsewhere, inter-individual differences in cognitive test performance may be attributable to differences that are specific to: (1) a given test, (2) a particular cognitive domain, and/or (3) the existence of a construct known as general cognitive ability [24, 57]. Interestingly, of the four neuropsychological tests used to assess cognitive performance in the French Three-City study, i.e., MMSE, Isaacs Set Test, Benton Visual Retention Test, and Free and Cued Selective Reminding Test, stricter adherence to a MED diet was associated with slower cognitive decline when measured using the MMSE, whereas no consistent association was found for any of the three remaining tests [7].

The present study had several limitations. First, the healthy-volunteer selection bias may partly explain why, in contrast to previous studies, no association between the diet score and performance in the VNR-test was found [49]. Second, the analyses did not consider the issue of total energy intake. Energy adjustment is a common method in nutritional epidemiology [58]. Thus, investigations of diet–disease relationships usually consider total energy intake as a confounding variable. In the present study, this was not possible, since the investigated dietary data were derived from a 29-item questionnaire on main food groups, which did not encompass total dietary intake. However, after adjustment for BMI, which may serve as a proxy of energy expenditure [11, 59], the results remained virtually unchanged. Third, the cognitive outcome was the result of the VNR-test, which measures fluid intelligence only [37]. Moreover, the VNR-test does not capture pathophysiological disorders, such as mild cognitive impairment or dementia, and does not reflect the impairments of cognitive performance that are characteristic of these conditions. Thus since fluid intelligence reflects only one cognitive domain, the results relating to the present diet score may not be transferable to other cognitive domains. Fourth, the cohort comprised middle-aged participants of Caucasian ancestry only. This demographic profile may limit the generalizability of the findings. However, the homogeneity of the study sample, and the resultant internal validity, may have limited potential confounding effects, such as SES, educational status, or ancestry. Finally, although the analyses controlled for a range of confounding factors, residual confounders may exist.

Since results from previous cohort studies of the association between diet/dietary pattern and cognitive function might have been inconclusive due to limited sample sizes, variation in age and gender distributions, and/or cross-study differences in phenotypic and exposure measurements, the present study also had several strengths. First, to our knowledge, it represents the largest single investigation of dietary patterns in relation to fluid intelligence to date. Second, all participants had undergone a uniform assessment of both their dietary intake and cognitive phenotype. Third, the comprehensive nature of the UK Biobank data enabled lifestyle factors such as educational status and SES to be taken into account. Fourth, the analyses considered genetic disposition, which is an advantage when investigating a multifactorial phenotype such as cognitive function.

In conclusion, no evidence was found for an association between fluid intelligence and a dietary score that considered fruit, vegetables, fish, processed meat, unprocessed meat, whole grain, and refined grain either at baseline or longitudinally. Results did not materially change after consideration of individual genetic disposition for cognitive function. Further studies, in particular long-term follow-up investigations, are warranted. Future research questions could be extended to more comprehensive or exploratory derived dietary patterns as well as the consideration of different cognitive domains.

## Supplementary Information

Below is the link to the electronic supplementary material.

Supplementary file1 (PDF 302 KB)

## Data Availability

Data described in the manuscript will not be made available, because we do not have the permission to share these data, but it can be applied at the UK Biobank resource at https://www.ukbiobank.ac.uk/enable-your-research/apply-for-access.

## Code availability

The code book and analytic code will be made available upon request pending application and approval.
