# Supplementary material for: Analysis of associations between dietary patterns, genetic disposition, and cognitive function in data from UK Biobank
Source: Eur J Nutr. 2022 Sep 24;62(1):511–21. doi: 10.1007/s00394-022-02976-y (PMC9899759; doi:10.1007/s00394-022-02976-y)
Supplement: Supplementary file 1 — Supplementary file1 (PDF 302 KB) [file 394_2022_2976_MOESM1_ESM.pdf]

Supplementary

Title: Analysis of associations between dietary patterns, genetic disposition, and cognitive function in data from UK Biobank

Authors

Christina-Alexandra Schulz, Leonie Weinhold, Matthias Schmid, Markus M. Nöthen, Ute Nöthlings

Christina-Alexandra Schulz, MPH, PhD

Institute of Nutrition and Food Sciences, Nutritional Epidemiology, University of Bonn, Bonn, Germany

E-mail: christina-alexandra.schulz@uni-bonn.de; Tel: +49 (0) 228 73 69857

Table S1 Coefficient estimates, P-values, and Cohen's f^2 between the variables included in the linear regression model used to investigate the association with the VNR-test results in 104,895 participants from the UK Biobank.

Variable	Coefficient estimate (95 %CI)	P-value	Cohen's f^2
Intercept	6.90 (6.80 to 7.00)		
Diet Score		0.610	0.00001
Intermediate diet score	-0.01 (-0.06 to 0.04)		
High diet score	-0.02 (-0.08 to 0.04)		
Age at recruitment	0.00 (0.00 to 0.00)	0.002	0.0001
Male sex	0.25 (0.23 to 0.27)	<0.001	0.004
Education		<0.001	0.17
upper secondary	-0.18 (-0.23 to -0.14)		
lower secondary	-1.00 (-1.03 to -0.97)		
vocational	-1.55 (-1.60 to -1.50)		
Other	-2.24 (-2.28 to -2.21)		
TD index		<0.001	0.003
Intermediate TD index	-0.07 (-0.10 to -0.05)		
High TD index	-0.3 (-0.34 to -0.26)		

Coefficient estimates (95% CI) and P-values were obtained from linear regression models. Abbreviations: CI=confidence interval; TD=Townsend deprivation index; VNR= Verbal-Numerical Reasoning.

Table S2 Associations between the individual dietary components and the VNR-test

	No fulfilment	yes	P value	*Cohen's f*^{2}
**Fruit**	no (n=49,315)	yes (n=55,580)		
Beta (95% CI)*	0 [Reference]	0.10 (0.08 - 0.13)	<0.0001	0.001
Beta (95% CI)**	0 [Reference]	-0.01 (-0.03 - 0.02)	6.64e-01	<0.001
**Vegetables**	no (n=14,050)	yes (n=90,845)		
Beta (95% CI)*	0 [Reference]	0.06 (0.03 – 0.01)	9.91e-04	<0.001
Beta (95% CI)**	0 [Reference]	-0.04 (-0.07 – -0.003)	3.19e-02	<0.001
**Fish**	no (n=48,617)	yes (n=56,278)		
Beta (95% CI)*	0 [Reference]	-0.06 (-0.08 - -0.03)	<0.0001	<0.001
Beta (95% CI)**	0 [Reference]	-0.09 (-0.11 - -0.06)	<0.0001	<0.001
**Processed meat**	no (n=33,599)	yes (n=71,296)		
Beta (95% CI)*	0 [Reference]	-0.11 (-0.13 - -0.08)	<0.0001	0.001
Beta (95% CI)**	0 [Reference]	-0.11 (-0.13 - -0.08)	<0.0001	0.001
**Unprocessed meat**	no (n=51,711)	yes (n=53,184)		
Beta (95% CI)*	0 [Reference]	0.04 (0.02 - 0.07)	8.16e-04	<0.001
Beta (95% CI)*	0 [Reference]	0.02 (0.0001 - 0.57)	4.84e-02	<0.001
**Whole grain**	no (n=94,985)	yes (n=9,910)		
Beta (95% CI)*	0 [Reference]	0.31 (0.26 - 0.35)	<0.0001	0.002
Beta (95% CI)**	0 [Reference]	0.15 (0.11-0.19)	<0.0001	0.001
**Refined grain**	no (n=32,238)	yes (n=72,657)		
Beta (95% CI)*	0 [Reference]	0.35 (0.32 - 0.38)	<0.0001	0.006
Beta (95% CI)**	0 [Reference]	0.14 (0.11-0.16)	<0.0001	0.001

**Coefficient estimates (95 %CI) and P-values were obtained from crude linear regression models.

**Coefficient estimates (95 %CI) and P-values were obtained from linear regression models adjusted for age, sex, education, and the Townsend deprivation index. Abbreviation: CI=confidence interval.

Table S3 Coefficient estimates and P-values of the variables included in the linear regression model used to investigate the change in the VNR-test results in 9,482 participants from the UK Biobank.

Variable	Coefficient estimate (95 %CI)	P-value
Intercept	6.73 (3.38 to 4.09)	
Baseline VNR-test result	0.59 (0.57 to 0.61)	<0.001
Diet Score		0.724
Intermediate diet score	-0.12 (-0.34 to 0.10)	
High diet score	0.10 (-0.32 to 0.51)	
Time to baseline	-0.05 (-0.08 to -0.01)	0.001
Age at recruitment	-0.01 (-0.01 to 0.00)	0.001
Male sex	0.14 (0.09-0.21)	<0.001
Education		<0.001
upper secondary	-0.09(-0.21 to -0.02)	
lower secondary	-0.47 (-0.55 to -0.39)	
vocational	-0.72 (-0.86 to -0.57)	
Other	-0.93 (-1.06 to -0.79)	
TD index		0.918
Intermediate TD index	0.93 (-1.06 to -0.07)	
High TD index	0.02 (-0.08 to -0.13)	
Diet Score : Time to baseline		0.423
Low diet score x time	0.01 (-0.02-0.05)	
Intermediate diet score x time	-0.02 (-0.08-0.05)	

Coefficient estimates (95% CI) and P-values were obtained from linear regression models. Abbreviations: CI=confidence interval; TD=Townsend deprivation index; VNR= Verbal-Numerical Reasoning.

Table S4 Association between fluid intelligence and the diet score or the PGS in 104,895 participants from the UK Biobank.

	n	Low	Intermediate	High	P-value	Cohen's f^2
**Diet score**	104895	0 [Reference]	0.16 (0.11-0.22)	0.25 (0.19-0.32)	<0.001	<0.001
**Diet score**[1]	104895	0 [Reference]	-0.01 (-0.06 - 0.04)	-0.02 (-0.08 - 0.04)	0.601	<0.001
**Diet score**[2]	100046	0 [Reference]	-0.01 (-0.06 - 0.05)	-0.02 (-0.08 - 0.04)	0.437	<0.001
**Diet score**[3]	104477	0 [Reference]	-0.01 (-0.06 - 0.05)	-0.02 (-0.08 - 0.04)	0.597	<0.001
**PGS**[4]	104895	0 [Reference]	1.55 (1.53-1.58)	3.36 (3.32-3.39)	<0.001	0.338
**PGS**[4]	104895	0 [Reference]	1.30 (1.27 - 1.32)	2.88 (2.84 - 2.91)	<0.001	0.262
**PGS**[5]	100046	0 [Reference]	1.29 (1.26 - 1.32)	2.87 (2.83 - 2.90)	<0.001	0.39
**PGS**[6]	104477	0 [Reference]	1.30 (1.27 - 1.32)	2.88 (2.85 - 2.91)	<0.001	0.39

Coefficient estimates (95% CI) and *P*-values were obtained from linear regression models
[1]adjusted for age, sex, education, and the Townsend deprivation index;
[2]adjusted for age, sex, education, the Townsend deprivation index, alcohol intake, smoking, and physical activity.
[3]adjusted for age, sex, education, the Townsend deprivation index, and BMI.
[4]adjusted for PC 1-20. [4]adjusted for age, sex, education, the Townsend deprivation index, and PC 1-20;
[5]adjusted for age, sex, education, the Townsend deprivation index, alcohol intake, smoking, physical activity and PC 1-20;
[6]adjusted for age, sex, education, the Townsend deprivation index, BMI, and PC 1-20.
Abbreviations: BMI=Bod mass index; CI=confidence interval; PGS= polygenic risk score; PC= principal component; PCA= principal component analysis.
